# Establishing the normal range of sperm DNA fragmentation index (% DFI) for rhesus macaques

**DOI:** 10.1038/s41598-023-46928-w

**Published:** 2023-11-16

**Authors:** Fernanda C. Burch, Philberta Y. Leung, Eric McDonald, Jared Jensen, Emily Mishler, Nadine Piekarski, Camilla M. Mendes, Andrew Sylwester, Carol B. Hanna

**Affiliations:** 1 Oregon National Primate Research Center, Oregon Health & Science University, Beaverton, OR USA; 2 Vaccine & Gene Therapy Institute, Oregon Health & Science University, Beaverton, OR USA; 3 College of Veterinary Medicine and Animal Science, University of São Paulo, São Paulo, SP Brazil

**Keywords:** Biological techniques, Medical research, Animal biotechnology

## Abstract

The Sperm Chromatin Structure Assay (SCSA) is a robust test with high repeatability and precision. It is a clinically accepted assay that defines risk for infertility in men by measuring the degree of DNA fragmentation (% DFI) in sperm. The objective of this study was to adapt and validate the SCSA for rhesus macaques (*Macaca mulatta*) and establish a range for % DFI in fertile males. Sperm samples from two different males were used to produce a % DFI validation curve before establishing a range using additional samples from n = 11 males. Sperm labeled with acridine orange were analyzed by flow cytometry to measure green fluorescence (native or intact DNA) and red fluorescence (fragmented DNA). Data were exported to FlowJo software to determine the % DFI for each sample. DNA fragmentation ranged from 0.1 to 2.4% DFI, with a mean ± SD = 1.1 ± 0.7% DFI (validation curve optimized to R^2^ > 0.95). In conclusion, we were able to successfully validate the SCSA in our institution and establish the first normal range for sperm DNA fragmentation in rhesus macaques. Our study provides a quantitative baseline for future evaluations to assess macaque fertility through the SCSA test.

## Introduction

The Sperm Chromatin Structure Assay (SCSA) was first introduced in 1980^1^ as an experimental method to determine DNA damage in sperm by using flow cytometry to measure susceptibility of in situ DNA denaturation. The initial protocol used brief exposure to high heat to induce denaturation of in situ DNA at sites of double-stranded and/or single-stranded DNA breaks^1^, and later optimized to utilize acid denaturation instead^2^. Acridine orange dye is used to differentiate native versus denatured DNA as it has an interesting property—when intercalated into native double stranded DNA, it fluoresces green (F 515–530 nm) when exposed to 488 nm light; when it stacks on single stranded nucleic acids (denatured DNA or RNA) it collapses into a crystal and undergoes a metachromatic shift from green to red fluorescence (F > 630 nm)^3,4^. The SCSA is a rapid, statistically robust test with high reliability, able to evaluate 5000 spermatozoa in less than 10 minutes^2^ with low intra-individual, intra-laboratory, and inter-laboratory variation. Although there are other tests that measure DNA fragmentation—such as the terminal deoxynucleotidyl transferase-mediated fluorescein-dUTP nick end labelling (TUNEL), the Comet Assay, the Sperm Chromatin Dispersion Assays (SCD; HALO)—these tests are not standardized and measure only 50–200 sperm by light microscopy (except for the TUNEL assay, which can also be done by flow cytometry)^5^. The lack of standardization of a single protocol makes it difficult to compare data and establish thresholds.

In contrast, the SCSA outperforms other assays in terms of efficiency, objectivity, and repeatability, and is now a globally accepted assay for clinical use to define risk of infertility in men based on the percentage of sperm with fragmented DNA (% DFI) ^5^. This index reflects sperm DNA integrity with high DFI values indicating a greater probability of infertility. The SCSA has been commercially used across many different species including men, boars, stallions, bulls^3,5^, mice^6^, bottle nose dolphins (*Tursiops aduncus*)^7^, cynomolgus macaques (*Macaca fascicularis*)^8^, and rhesus macaques (*Macaca mulatta*)^9^. It is a useful diagnostic tool in research to assess deleterious effects on sperm in a broad spectrum of studies such as toxicology, air pollution, and reproductive biology^2^. The percent DNA fragmentation index threshold used to identify reduced fertility has only been established for a few species, including men (% DFI > 25–30%), boars (% DFI > 6%), bulls (% DFI > 10–20%), and stallions (% DFI > 28%)^3,5^.

There are only two reports on the use of SCSA in macaques. Both studies use a control group of animals to roughly estimate the normal range of % DFI for comparison. The first study evaluated the effect of chronic lead treatment on sperm quality in cynomolgus macaques^8^. The study showed that there were no negative impacts on traditional measures of semen quality, such as concentration, viability, and motility. However, there was a significant difference in the amount of sperm with fragmented DNA. The authors concluded that the SCSA is a more sensitive assay for toxicant-induced effects on semen quality when compared to traditional measures^8^. The second study evaluated the effects of moderate calorie restriction on semen characteristics in rhesus macaques^9^. The % DFI was 8.5 ± 8.0% for the control group and 1.9 ± 1.1% for the calorie restriction group, with no significant difference between the two groups^9^. Given the low sample size (n = 10–12 per group) and the high standard deviation observed in the control group, the results are difficult to interpret. Therefore, the objective of this study was to validate the SCSA for rhesus macaques (*Macaca mulatta*) and determine the normal range of % DFI for males of this species with established fertility.

## Results

### Validation of the assay

**Figure 1:**
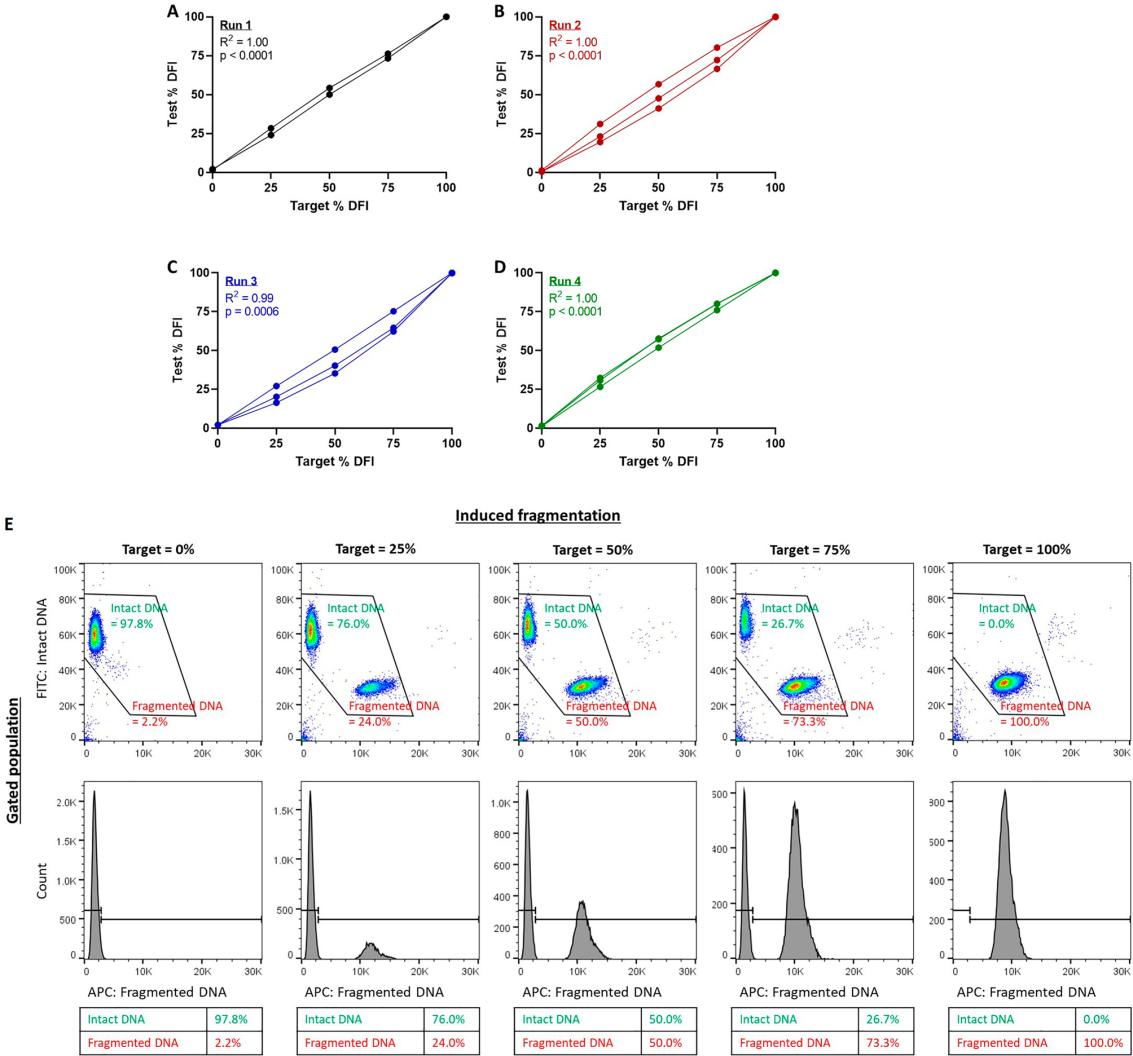
Successful validation of the SCSA assay in rhesus macaques. In four separate runs, we used sperm samples from N = 2–3 different males. A positive control was obtained by treating sperm with strong acid detergent (1.2 M HCl) to induce 100% DFI, while negative controls were obtained via standard treatment (0.08 M HCl; **E**). Positive and negative samples were mixed at 3 different concentrations (1:3, 1:1, and 3:1, **E**). Within the FlowJo software, debris and dead cells were gated out. The resulting gated population was converted into a frequency histogram with APC (red fluorescence; fragmented DNA) on the X-axis. Cells with high red fluorescence were considered as cells with fragmented DNA and included in the percent DFI. In all four runs, we were able to produce a curve with R^2^ ≥ 0.95, the minimum standard for a valid assay (**A–D**).

The assay was initially validated using samples from two different males to measure the % DFI by flow cytometry. The validation curve generated had an R^2^ = 1.0 (Fig. 1A), higher than R^2^ = 0.95, our minimum standard for a valid assay (see Materials and Methods). To obtain further validation of the protocol, we ran three additional experiments, with three unique males, and confirmed that each of the validation curves reached an R^2^ ≥ 0.95 (Figs. 1B–D). Figure 1E shows an example of the results obtained in FlowJo used to generate one of the validation curves.

**Figure 2:**
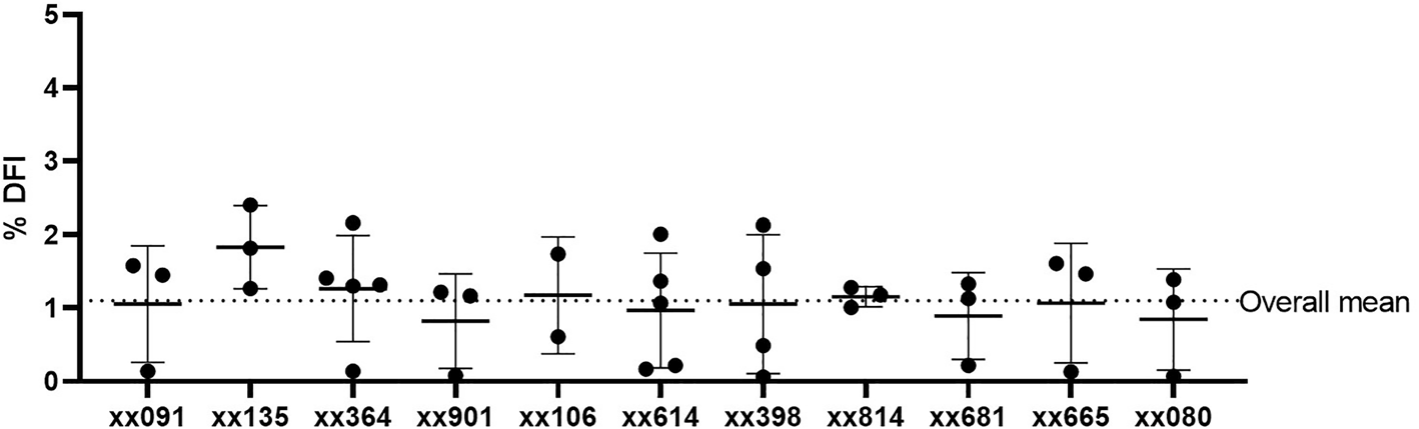
Range of sperm DNA fragmentation Index (% DFI) in 11 adult male rhesus macaques. Data shown as mean ± SD. Each dot represents a sample from one semen collection. Dotted line indicates the overall mean for all 11 males.

**Figure 3:**
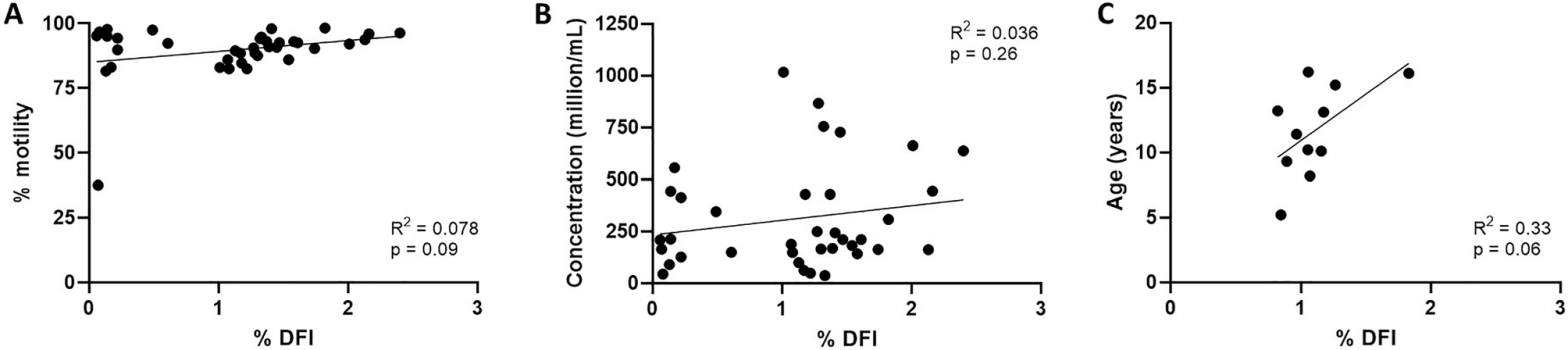
Correlation of the DNA fragmentation index (% DFI) with sperm motility (**A**), concentration (**B**), and age (**C**). Each dot represents a sample from one semen collection. The average % DFI for each male was used in (**C**).

After validation, we collected 2–6 samples from N = 11 males for a total of 37 samples. Figure 2 shows each data point (black dots), as well as the mean % DFI ± standard deviation for each male and the overall mean for all males. As summarized in Table 1, we established the normal range (min–max) of DNA fragmentation index for rhesus macaques to be 0.1–2.4% with a mean ± standard deviation (SD) of 1.1 ± 0.7%. Additional CASA data are provided as supplemental data. We further show that there was no correlation between % DFI and motility, concentration, or age (Fig. 3A–C), indicating that % DFI was independent of these variables.

**Table 1:** Sperm DNA fragmentation Index (% DFI) in healthy rhesus macaques.

ID #	n	% DFI		Motility (%)		Concentration (million/mL)		Age (years)
		Range (min–max)	Mean ± SD	Range (min–max)	Mean ± SD	Range (min–max)	Mean ± SD	
xx091	3	0.1–1.6	1.1 ± 0.8	90.9–95.1	93.0 ± 2.1	142–726	360 ± 319	16
xx135	3	1.3–2.4	1.8 ± 0.6	90.6–98.3	95.1 ± 4.1	249–636	398 ± 209	16
xx364	5	0.1–2.2	1.3 ± 0.7	87.6–98.0	94.7 ± 4.3	165–754	410 ± 229	15
xx901	3	0.1–1.2	0.8 ± 0.6	88.5–96.8	89.3 ± 7.2	44–61	52 ± 9	13
xx106	2	0.6–1.7	1.2 ± 0.8	90.4–92.4	91.4 ± 1.4	149–163	156 ± 10	13
xx614	5	0.2–2.0	1.0 ± 0.8	83.1–93.1	88.8 ± 4.2	188–661	449 ± 178	11
xx398	4	0.1–2.1	1.1 ± 0.9	86.0–97.5	93.2 ± 5.0	161–344	224 ± 83	10
xx814	3	1.0–1.3	1.2 ± 0.1	83.0–88.6	85.4 ± 2.9	428–1016	771 ± 305	10
xx681	3	0.2–1.3	0.9 ± 0.6	89.5–94.8	92.9 ± 3.0	36–126	87 ± 46	9
xx665	3	0.1–1.6	1.1 ± 0.8	81.6–92.6	88.9 ± 6.4	90–210	170 ± 70	8
xx080	3	0.1–1.4	0.8 ± 0.7	37.6–91.0	70.4 ± 28.7	148–168	160 ± 10	5
Total	37	0.1–2.4	1.1 ± 0.7	37.6–98.3	89.7 ± 10.1	36–1016	311 ± 248	5–16

Semen samples were obtained from 11 adult males and analyzed with the Sperm Chromatin Structure Assay (SCSA) to determine the % DFI. SD = standard deviation.

## Discussion

This study is the first to suggest a normal range of 0.1–2.4% DFI in fertile rhesus macaque males with a mean ± SD of 1.1 ± 0.7%. According to the WHO laboratory manual^12^, for an average of 1% for a given sperm parameter, the acceptable difference between replicates is 2% based on the rounded 95% confidence interval. Based on that information, our results showed a very low intra- and inter-individual variability, suggesting that the % DFI is consistently low in fertile rhesus macaques. The mean obtained in our study (1.1 ± 0.7%) was similar to that obtained by Sitzmann et al. in their treated group (1.9 ± 1.1%)^9^. In said study, the control group was fed specially formulated biscuits supplemented with daily fresh fruits and vegetables, while the treated group was fed the same diet, but with a 30% calorie restriction. There was no statistical difference between the two groups for any of the traditional ejaculate characteristics investigated (i.e. ejaculate weight, volume, osmolarity, and pH, sperm count, concentration, motility, viability, and morphology). Moreover, there was also no statistical difference in the % DFI between the two groups (8.5 ± 8.0%—control group and 1.9 ± 1.1%—treated group) and the authors attributed the apparent difference to the presence of an outlier with 32.5% DFI. Despite being an outlier, this datapoint was not removed from their analysis because it would reduce their n from four to three males^9^. When compared to humans, however, the degree of DNA fragmentation for fertile rhesus males was much lower than for fertile men. The % DFI values for men that produce a pregnancy within either 3 months or 4–12 months, are 11.2% and 15.5%, respectively^2^. On the other hand, a % DFI > 25% is considered the clinical threshold for infertility in men, suggesting that the ART lab should consider fertilization by intracytoplasmic sperm injection (ICSI)^4^.

In men, it has been shown that % DFI is positively correlated with semen volume and the proportion of sperm with abnormal head morphology and it is negatively correlated with sperm concentration, progressive motility, total motility, and % normal sperm morphology^10^. Since the percentage of sperm with normal morphology is higher in rhesus macaques when compared to men (57.8 ± 26% in rhesus macaques^11^ versus 3–5% in humans^12^), it makes sense that the degree of DNA fragmentation would also be lower in the rhesus macaque.

At the time of this study, we were unable to show any correlations with infertility parameters as we did not have access to infertile or subfertile males. Studies of this nature will be performed as candidate animals are identified. Further investigation is needed to establish the threshold of % DFI to determine infertility in rhesus macaques, as well as determine the effects of cryopreservation on the % DFI in this species. Once we identify infertile males in our colony (i.e. males that are unable to sire infants), we will add them to our pool to establish a fertility threshold for rhesus macaques. This data will be useful for future projects evaluating fertility, toxicology, reproductive biology, and others. In conclusion, we were able to successfully validate the SCSA in our institution. Moreover, our study provides a quantitative baseline for evaluating changes in macaque fertility through flow cytometry analysis.

## Methods

### Animals

All animals used in this study were Indian-origin rhesus macaques housed indoors at the Oregon National Primate Research Center (ONPRC), an AAALAC-accredited facility. Monkeys were provided with a 12-h light cycle (07:00–19:00) year-round with natural humidity and temperatures were kept between 7 °C (45°F) and 30 °C (85°F), per the Animal Welfare Act. All rhesus macaques were fed a commercial monkey chow twice daily (6–7 biscuits/meal according to weight; LabDiet 5000, Purina Mills, Saint Louis, MS, USA) and given fresh produce or other food enrichment daily. Animals had ad libitum access to water. Additionally, the monkeys’ cognitive welfare was supported by the ONPRC Behavioral Management Plan which includes enrichment items such as toys, foraging devices and enrichment, frozen treats, TV, radio, mirrors, and removable verandas.

The study included 11 adult males (5 to 16 years old and weight 7.4–14.2 kg) that were part of the Assisted Reproductive Technologies (ART) Core pool of males. Therefore, these males had proven fertility through ongoing IVF studies. All macaques were born at the ONPRC and were considered specific pathogen free (SPF) for simian retrovirus, simian immunodeficiency virus, simian T-cell leukemia virus, and macacine herpesvirus 1. They were given a clinical health evaluation prior to sample collection and monitored throughout the study, with no evidence of pre-existing or acquired disease. All males were housed in pairs, unless the Behavioral Services Unit deemed them incompatible with other monkeys. All animal procedures were pre-approved by the Institutional Animal Care and Use Committee at the Oregon Health & Science University, followed the National Institute of Health’s Guide for the Care and Use of Laboratory Animals, and complied with the ARRIVE guidelines.

### Reagents

All reagents used to prepare working solutions were purchased from Sigma, unless otherwise stated. All staining solutions and buffers for the SCSA® were prepared as described by Evenson (2011)^3,4^.

### Semen collection and processing

All semen collections were performed by penile electroejaculation without sedation using a PTE 110 Volt AC electroejaculator (P–T Electronics, Model 303, Boring, OR) as previously described^13^. Ejaculate was collected directly into a wide mouth container and sat at 37 °C for 30 min to allow for liquefaction. The liquid portion of the ejaculate was transferred into a 15 mL conical tube. The coagulated plug was washed with 3 mL of TALP-HEPES (supplemented with 3 mg/ml of bovine serum albumin)^14^ to recover remaining spermatozoa then combined with the liquid fraction before adding additional TALP-HEPES Q.S. to 12 mL. Samples were washed twice by centrifugation at 300×*g* for 7 min at 37 °C. After removal of the supernatant (11 mL), the sperm pellet was resuspended in the remaining 1 mL of TALP-HEPES and analyzed by a computer assisted sperm analysis (CASA) system (IVOS II-Animal Motility software, version 1.11, Hamilton Thorne, Beverly, MA) for sperm concentration and motility^13^. After analysis, aliquots containing 250,000 spermatozoa were placed in separate 0.6 ml centrifuge tubes and snap frozen in liquid nitrogen for storage in a − 80 °C freezer until analysis. On the day of analysis, samples were thawed in a water bath at 37 °C for 30 s.

### Validation of the assay

We initially ran a test to generate a validation curve as described previously^15^. Samples were processed as described above and aliquoted into four separate tubes—two tubes were processed as positive controls and the other two were processed as negative controls. The negative control samples underwent the standard treatment, as follows. Each sample was mixed with 150 µl of TNE buffer (Tris-Hydrochloric acid 0.01 M, NaCl 0.15 M, EDTA 1 mM; pH 7.4) and 300 µl of acid detergent (HCl 0.08 M, NaCl 0.15 M, Triton X-100 0.1%; pH 1.2), then incubated for 30 s before adding 900 µl of acridine orange solution (Citric acid 0.1 M, Na_2_HPO_4_ 0.2 M, EDTA 0.001 M, NaCl 0.15 M, Acridine orange 6 µg/ml in Millipore water; pH 6.0). The positive control samples were initially treated with 300 µl of strong acid detergent (HCl 1.2 M, pH 0.1) for 1 min to induce 100% DFI, followed by 150 ul of TNE buffer. The samples were then centrifuged at 200×*g* for 5 min, after which the supernatant (350 µl) was removed and 900 µl of AO were added. The positive and negative control samples were mixed at three different ratios 1:3, 1:1, 3:1 to produce a curve; the assay was deemed valid only if R^2^ ≥ 0.95.

### Percent DNA fragmentation index (% DFI)

After initial validation of the assay, semen samples were collected from each male (N = 11) to determine a reference range of DNA fragmentation index in rhesus macaques. The samples underwent the standard treatment described above for the negative control. The samples were run through a flow cytometer (BD LSR II Cell Analyzer, BD Biosciences, San Jose, CA 95,131) using a 488 nm excitation wavelength. Fluorescence of individual cells was collected using a FITC filter (505–525 nm) to measure green fluorescence (native or intact DNA) and an APC filter (650–670 nm) to measure red fluorescence (broken DNA). Raw data were exported to FlowJo (version 10.8.1; FlowJo, LLC; Ashland, OR 97,520). After gating out debris and dead cells, a frequency histogram (with red fluorescence, APC, on the X-axis) was used to determine the % DFI, the percent of total sperm cells that had high red fluorescence^3^.

### Statistical analysis

Statistical analyses were conducted using Graphpad Prism version 9.3.1. Data were reported as mean ± standard deviation (SD). Linear regressions were performed on the validation curve data.

### Supplementary Information

Supplementary Information.

## Data Availability

The datasets generated during and/or analyzed during the current study are available from the corresponding author on reasonable request.
