# Supplementary material for: Establishing the normal range of sperm DNA fragmentation index (% DFI) for rhesus macaques
Source: Sci Rep. 2023 Nov 16;13:20016. doi: 10.1038/s41598-023-46928-w (PMC10654681; doi:10.1038/s41598-023-46928-w)
Supplement: Supplementary file 1 — Supplementary Information. [file 41598_2023_46928_MOESM1_ESM.docx]

**Supplemental Table 1a.** Computer assisted sperm analysis (CASA) data from n=11 rhesus macaques (*Macaca mulatta*). Data shown as mean ± standard deviation.

| **ID #** | **n** | **Total sample (million)** | **Total concentration (million/mL)** | **Motile concentration (million/mL)** | **% Motile** | **Progressive concentration (million/mL)** | **% Progressive** | **Slow concentration (million/mL)** | **% Slow** |
| --- | --- | --- | --- | --- | --- | --- | --- | --- | --- |
| xx091 | 3 | 164.7 ± 41.8 | 360.3 ± 319.0 | 331.4 ± 286.7 | 93.0 ± 2.1 | 290.3 ± 257.4 | 80.7 ± 1.5 | 2.7 ± 2.6 | 0.8 ± 0.4 |
| xx135 | 3 | 398.2 ± 208.7 | 398.2 ± 208.7 | 380.9 ± 205.1 | 95.1 ± 4.0 | 286.0 ± 125.4 | 73.7 ± 7.7 | 7.7 ± 10.6 | 1.5 ± 1.5 |
| xx364 | 5 | 296.9 ± 137.0 | 410.1 ± 228.6 | 390.7 ± 217.4 | 94.7 ± 4.3 | 301.3 ± 175.2 | 72.6 ± 8.2 | 6.3 ± 3.4 | 1.8 ± 1.4 |
| xx901 | 3 | 51.5 ± 9.1 | 51.5 ± 9.1 | 45.8 ± 7.6 | 89.3 ± 7.2 | 33.5 ± 2.7 | 65.7 ± 6.0 | 0.6 ± 0.5 | 1.1 ± 1.0 |
| xx106 | 2 | 156.2 ± 9.7 | 156.2 ± 9.7 | 142.7 ± 6.7 | 91.4 ± 1.4 | 118.0 ± 10.1 | 75.5 ± 1.7 | 2.4 ± 0.7 | 1.5 ± 0.3 |
| xx614 | 5 | 449.5 ± 177.7 | 449.5 ± 177.7 | 400.7 ± 162.5 | 88.8 ± 4.3 | 284.2 ± 100.9 | 64.2 ± 4.5 | 19.5 ± 7.6 | 4.5 ± 1.1 |
| xx398 | 4 | 229.5 ± 100.4 | 229.5 ± 100.4 | 214.7 ± 105.4 | 92.3 ± 5.9 | 164.2 ± 76.7 | 71.1 ± 3.4 | 11.5 ± 3.6 | 5.7 ± 2.9 |
| xx814 | 3 | 291.4 ± 118.8 | 770.6 ± 305.5 | 658.4 ± 258.4 | 85.4 ± 2.9 | 455.1 ± 227.4 | 56.7 ± 10.4 | 43.6 ± 6.6 | 6.3 ± 2.6 |
| xx681 | 3 | 87.4 ± 46.0 | 87.4 ± 46.0 | 80.9 ± 42.8 | 92.9 ± 3.0 | 67.0 ± 37.0 | 78.0 ± 11.5 | 1.7 ± 0.7 | 2.1 ± 0.5 |
| xx665 | 3 | 150.3 ± 85.1 | 150.3 ± 85.1 | 134.6 ± 85.1 | 87.6 ± 7.1 | 111.6 ± 66.7 | 73.6 ± 2.8 | 2.6 ± 3.0 | 1.4 ± 1.3 |
| xx080 | 3 | 160.4 ± 10.2 | 160.4 ± 10.2 | 112.5 ± 46.6 | 70.4 ± 28.7 | 83.4 ± 53.5 | 52.2 ± 32.9 | 12.5 ± 11.4 | 7.7 ± 6.9 |
| **Total** | **37** | 242.7 ± 163.9 | 316.7 ± 253.9 | 285.3 ± 228.5 | 89.4 ± 10.3 | 215.0 ± 170.3 | 69.1 ± 12.8 | 10.9 ± 12.9 | 3.2 ± 3.1 |

**Supplemental Table 1b**. Computer assisted sperm analysis (CASA) data from n=11 rhesus macaques (*Macaca mulatta*). Data shown as mean ± standard deviation. ALH = amplitude of lateral head displacement (µm); BCF = beat cross frequency (Hz); DAP = distance average path (µm); WOB = wobble (average path velocity (VAP) / curvilinear velocity (VCL), %); DCL = curvilinear distance (µm); DSL = straight line distance (µm); LIN = linearity (straight line velocity (VSL) / curvilinear velocity (VCL), %); STR = straightness (straight line velocity (VSL) / average path velocity (VAP), %).

| **ID #** | **n** | **Static concentration (million/mL)** | **% Static** | **Motile mean ALH** | **Motile mean BCF** | **Motile mean DAP** | **Motile mean WOB** | **Motile mean DCL** | **Motile mean DSL** | **Motile mean LIN** | **Motile mean STR** |
| --- | --- | --- | --- | --- | --- | --- | --- | --- | --- | --- | --- |
| xx091 | 3 | 28.9 ± 32.4 | 7.0 ± 2.1 | 8.6 ± 1.1 | 34.9 ± 1.6 | 145.5 ± 25.9 | 75.1 **±** 5.2 | 190.5 ± 22.4 | 128.7 ± 24.4 | 68.0 ± 5.8 | 89.7 ± 2.3 |
| xx135 | 3 | 17.3 ± 10.5 | 4.9 ± 4.0 | 7.4 ± 0.1 | 40.8 ± 2.8 | 175.8 ± 36.9 | 79.8 ± 1.5 | 218.6 ± 46.6 | 145.7 ± 34.5 | 69.4 ± 3.8 | 86.0 ± 3.8 |
| xx364 | 5 | 19.4 ± 15.2 | 5.3 ± 4.3 | 8.2 ± 2.4 | 35.2 ± 3.1 | 152.0 ± 41.8 | 75.7 ± 9.3 | 193.2 ± 32.0 | 129.1 ± 36.9 | 66.4 ± 9.4 | 86.4 ± 2.5 |
| xx901 | 3 | 5.7 ± 3.8 | 10.7 ± 7.2 | 10.3 ± 2.2 | 30.5 ± 2.6 | 100.9 ± 14.8 | 62.2 ± 2.2 | 162.1 ± 30.6 | 83.3 ± 16.9 | 53.4 ± 3.0 | 85.1 ± 3.4 |
| xx106 | 2 | 13.5 ± 3.0 | 8.6 ± 1.4 | 7.0 ± 1.1 | 39.7 ± 1.3 | 150.7 ± 18.7 | 73.5 ± 6.1 | 199.7 ± 3.1 | 133.2 ± 21.5 | 66.3 ± 7.1 | 88.6 ± 1.6 |
| xx614 | 5 | 48.8 ± 27.1 | 11.2 ± 4.3 | 6.8 ± 1.1 | 38.1 ± 0.8 | 147.8 ± 12.9 | 77.1 ± 1.5 | 185.6 ± 14.6 | 123.9 ± 14.6 | 66.8 ± 2.7 | 83.9 ± 2.5 |
| xx398 | 4 | 14.9 ± 9.4 | 7.7 ± 5.9 | 6.5 ± 0.6 | 40.3 ± 1.0 | 167.6 ± 24.9 | 74.8 ± 3.2 | 215.2 ± 29.6 | 144.4 ± 17.2 | 66.3 ± 2.2 | 87.0 ± 0.9 |
| xx814 | 3 | 112.2 ± 54.8 | 14.6 ± 2.9 | 6.6 ± 0.3 | 39.4 ± 0.4 | 133.4 ± 34.3 | 67.3 ± 8.6 | 181.4 ± 27.6 | 113.7 ± 32.6 | 57.2 ± 9.6 | 81.2 ± 4.6 |
| xx681 | 3 | 6.5 ± 4.3 | 7.1 ± 3.0 | 6.3 ± 0.8 | 43.1 ± 3.0 | 171.4 ± 29.2 | 74.4 ± 6.6 | 227.0 ± 24.9 | 151.8 ± 26.8 | 67.1 ± 8.0 | 88.9 ± 5.3 |
| xx665 | 3 | 15.6 ± 0.0 | 12.4 ± 7.1 | 6.0 ± 0.8 | 41.8 ± 1.1 | 184.2 ± 4.2 | 80.9 ± 0.3 | 227.5 ± 4.4 | 162.8 ± 1.0 | 73.5 ± 1.9 | 90.0 ± 2.3 |
| xx080 | 3 | 47.9 ± 47.5 | 29.6 ± 28.7 | 5.4 ± 1.3 | 41.1 ± 0.4 | 138.8 ± 77.5 | 69.3 ± 16.6 | 180.8 ± 80.6 | 122.6 ± 71.0 | 61.3 ± 17.7 | 84.7 ± 7.6 |
| **Total** | **37** | 31.4 ± 36.8 | 10.6 ± 10.3 | 7.3 ± 1.8 | 38.3 ± 3.9 | 150.6 ± 36.4 | 73.8 ± 8.0 | 196.41 ± 35.4 | 129.3 ± 33.3 | 65.0 ± 8.4 | 86.2 ± 4.0 |

**Supplemental Table 1c.** Computer assisted sperm analysis (CASA) data from n=11 rhesus macaques (*Macaca mulatta*). Data shown as mean ± standard deviation.

| **ID #** | **n** | **Total mean area** | **Total mean elongation** | **% Bent tail** | **% Coiled tail** | **% Distal droplet** | **% Proximal droplet** |
| --- | --- | --- | --- | --- | --- | --- | --- |
| xx091 | 3 | 18.0 ± 0.6 | 0.4 ± 0.1 | 2.0 ± 1.3 | 0.2 ± 0.3 | 2.0 ± 0.9 | 37.0 ± 10.3 |
| xx135 | 3 | 19.3 ± 0.2 | 0.5 ± 0.0 | 0.7 ± 0.4 | 0.1 ± 0.1 | 4.2 ± 1.9 | 32.9 ± 4.2 |
| xx364 | 5 | 19.2 ± 0.7 | 0.4 ± 0.1 | 0.9 ± 0.6 | 0.3 ± 0.4 | 2.1 ± 0.5 | 43.8 ± 14.3 |
| xx901 | 3 | 18.7 ± 0.8 | 0.4 ± 0.1 | 0.6 ± 0.5 | 0.6 ± 1.1 | 1.6 ± 0.5 | 47.3 ± 24.8 |
| xx106 | 2 | 18.5 ± 1.0 | 0.4 ± 0.1 | 1.1 ± 0.7 | 0.3 ± 0.0 | 4.5 ± 2.3 | 49.1 ± 10.5 |
| xx614 | 5 | 19.9 ± 0.3 | 0.4 ± 0.0 | 1.2 ± 0.6 | 0.3 ± 0.2 | 4.9 ± 1.7 | 44.6 ± 4.8 |
| xx398 | 4 | 19.6 ± 0.7 | 0.5 ± 0.1 | 2.3 ± 2.0 | 1.2 ± 1.0 | 4.6 ± 0.2 | 33.2 ± 12.5 |
| xx814 | 3 | 19.5 ± 0.8 | 0.5 ± 0.0 | 2.1 ± 1.8 | 0.7 ± 0.4 | 5.4 ± 1.0 | 44.7 ± 1.0 |
| xx681 | 3 | 19.0 ± 0.8 | 0.4 ± 0.1 | 1.4 ± 1.3 | 0.3 ± 0.3 | 1.0 ± 1.4 | 49.3 ± 7.6 |
| xx665 | 3 | 18.6 ± 0.3 | 0.5 ± 0.0 | 1.7 ± 0.1 | 0.0 ± 0.0 | 4.4 ± 2.5 | 48.7 ± 9.3 |
| xx080 | 3 | 18.6 ± 0.6 | 0.4 ± 0.1 | 4.6 ± 4.1 | 2.1 ± 1.3 | 4.7 ± 2.1 | 51.9 ± 19.2 |
| **Total** | **37** | 19.1 ± 0.8 | 0.5 ± 0.1 | 1.6 ± 1.7 | 0.6 ± 0.8 | 3.4 ± 2.0 | 43.6 ± 12.2 |
